# Key Factors Influencing Gelation in Plant vs. Animal Proteins: A Comparative Mini-Review

**DOI:** 10.3390/gels10090575

**Published:** 2024-09-03

**Authors:** Mohammadreza Khalesi, Kyeesha Glenn-Davi, Nima Mohammadi, Richard J. FitzGerald

**Affiliations:** Department of Biological Sciences, University of Limerick, V94 T9PX Limerick, Irelandnima.mohammadi@ul.ie (N.M.); dick.fitzgerald@ul.ie (R.J.F.)

**Keywords:** gelation, anti-nutritional factors, protein structure, pH sensitivity, ionic strength

## Abstract

This review presents a comparative analysis of gelation properties in plant-based versus animal-based proteins, emphasizing key factors such as pH, ionic environment, temperature, and anti-nutritional factors. Gelation, a crucial process in food texture formation, is influenced by these factors in varying ways for plant and animal proteins. Animal proteins, like casein, whey, meat, and egg, generally show stable gelation properties, responding predictably to pH, temperature, and ionic changes. In contrast, plant proteins such as soy, pea, wheat, and oilseed show more variable gelation, often requiring specific conditions, like the presence of NaCl or optimal pH, to form effective gels. Animal proteins tend to gel more reliably, while plant proteins require precise environmental adjustments for similar results. Understanding these factors is crucial for selecting and processing proteins to achieve desired textures and functionalities in food products. This review highlights how changing these key factors can optimize gel properties in both plant- and animal-based proteins.

## 1. Introduction

Proteins are generally classified based on their solubilities [1]. Protein solubility is defined as the amount of protein that can be dissolved into an aqueous solution. Soluble proteins are characterized by a high degree of surface hydrophilicity and a high charge of surface amino acids (AAs). A major factor influencing the solubility is the composition of AAs on the surface of the protein, which controls interactions with other nearby proteins or the solvent. AAs are generally classified into three categories, nonpolar, uncharged polar, and charged polar. The electronegativity of the R-chain side group of the AA determines its behavior around other proteins or solvents. The solubility of a protein can be altered by changing the pH, ionic strength, or temperature of the solution [2]. Knowledge of the solubility of a specific protein will inform whether it will have good emulsification, foaming, thickening, and gelation properties. Additionally, it is important to understand the solubility of proteins in liquid food products (e.g., beverages, soups).

Understanding the gelation properties of proteins is important for the production of many food products, including yogurt, cheese, and confectionary items. Milk, egg, and animal proteins are popular gelling agents due to their availability, cost-effectiveness, and favorable gelling characteristics. With the rising trend toward plant-based diets, there is a growing interest in plant-based proteins derived from legumes, grains, and oilseeds as promising alternatives for gelation [3,4].

**Figure 1:**
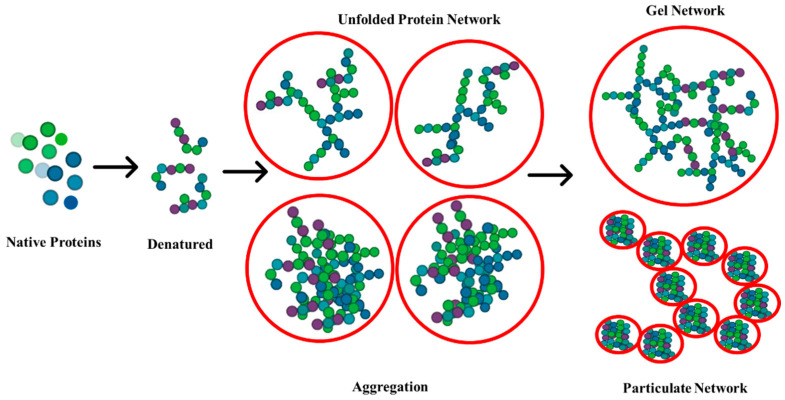
Illustration of protein denaturation and network formation: native proteins in their folded, functional state; denatured proteins undergoing unfolding and aggregation; formation of unfolded protein networks and aggregates; development of gel and particulate networks after denaturation.

The gelling properties of proteins give texture to foods. The formation of a three-dimensional network through protein–protein and protein–solvent interactions results in a semisolid, viscoelastic structure. Depending on their composition, gels can vary in firmness, opacity, and elasticity. Gels can be categorized based on the arrangement of molecules (random aggregates vs. string of beads), as shown in Figure 1, and on their visual appearance (opaque vs. transparent). There are two main ways gels are formed in food systems: heat-induced and cold gelation. These treatments promote protein denaturation, which exposes amino acid residues that then participate in covalent or noncovalent cross-linking interactions. The formation of a gel depends on several environmental conditions, such as pH, ionic strength, and heating [5].

Given the diverse applications of protein gels in the food industry and the increasing interest in plant-based proteins, it is vital to compare the gelation properties of both plant-based and animal-based proteins. Previous review articles on “animal proteins × plant proteins” have primarily focused on advancements in technology and health benefits of plant-based proteins [6]; various extraction methods, sources, and applications [7]; gelling properties of different animal and plant proteins [8,9,10]; general nutritional differences and functional properties [11]; plant-based protein–polysaccharide interactions [12]; and protein stabilization in multiphase systems [13]. However, none of these studies focus on comparing the gelation properties of plant-based versus animal-based proteins, considering factors like pH, ionic environment, and temperature. Our study aims to fill this gap by investigating these factors across the various scientific literatures, informing better protein selection and processing strategies, and ultimately enhancing the development of food products with the desired texture and consistency.

## 2. Effect of pH on Gelation Properties

The pH significantly affects the gelation properties of proteins as well as their structural and functional characteristics. During gelation, proteins exhibit different behaviors depending on the pH at which they are processed. For example, proteins gelled at a higher pH form stronger, more homogeneous gels, while those gelled at a lower pH tend to form weaker, more heterogeneous structures that may leak water [14]. The isoelectric point (pI) is the pH at which a molecule has no net charge and plays a key role in determining the swelling behavior and density of the gel network [15]. Most proteins are least soluble at their pI, and altering the pH can increase their solubility [5]. The dynamic rheological properties of proteins during heat-induced gelation are also pH dependent, with optimal gelation occurring at specific pH levels [16]. Hence, changing the pH is a critical strategy to optimize performance in various applications, including food processing and biopolymer development [17].

### 2.1. Animal Proteins

Sodium caseinate (NaCN) and rennet casein gels were formed by preparing a dispersion followed by the addition of glucono delta-lactone (GDL) at 4 °C in quantities sufficient to bring the pH to 4.6. The gel point was determined using a rheometer to measure the change in storage modulus (G′). The gel point was defined as the pH at which G′ ≥ 1.0 Pa. At 5% (*w*/*w*) protein, NaCN formed gels after 60 min at pH 5.03, with a final G′ of 403 Pa [18]. The impact of altering the pH between 5.2 and 6.7 on the heat-induced gelation of casein micelles (CMs) was investigated by measuring the dynamic elastic properties. The CMs were in the form of a native phospho-caseinate powder (NPCP), and gel formation was induced by heating the solution at 50 °C for 16 h. The results showed that the stiffness of the gel was dependent on the protein concentration but independent of the heating temperature and pH. This study compared the gelation of NaCN and CMs, concluding that the rate of gelation of NaCN increased with increasing temperature and decreasing pH [19]. Summaries of these results are shown in Table 1.

The rheological properties of heat-set gels of whey protein isolate (WPI) and whey protein concentrate (WPC) were compared under varying temperatures, protein concentrations, pHs, and salt concentrations. Gels were formed with 11.25% protein and heated at 75 °C for 45 min. Since WPI contains lower levels of fats than WPC, it resulted in clearer gels at pH 2–3 and pH 7–8, whereas WPC gels were opaque across all pH values. In terms of gel strength, the lowest values were observed near the isoelectric point (pI) of the whey proteins, at which the net charge is negligible and low levels of intermolecular interactions occur. The highest gel strength for WPC and WPI was observed at pH 8.0 and 6.0, respectively. It can be concluded that pH does affect the gelation properties of whey proteins; however, the results vary based on the type of protein used [21]. Another study found that the gel strength of heat-induced WPI gels varied with the pH (2, 5.6, and 7). The acidic and neutral pH values provided the most desirable gelling characteristics, which were defined by a regular structure and good stability [22].

The heat-induced gelation of mucin-free egg white was investigated through rheological measurements. Solutions were heated across a temperature range of 50–90 °C for 16 min. Egg white protein gels formed a coarse structure near their pI (pH 5) and exhibited low viscoelastic properties and low water-holding capacity (WHC) [29]. Similarly, researchers reported that at pH 4.5, close to the isoelectric point of ovalbumin, the gels became brittle and less firm [30]. Decreasing the pH notably reduced WHC of the egg white gels. At higher pH levels of 7 and 9, the gels were more viscoelastic and had higher WHC [29]. Decreasing the pH notably reduced the WHC of the EW gels. Another study found that increasing pH values (pH 2, 5, 8) of whole egg, egg white, and egg yolk proteins raised their gelation point (°C). The texture profile analysis revealed that whole egg and egg yolk had the highest hardness at pH 8 and the lowest hardness at pH 2, while egg whites exhibited the highest hardness at pH 5 and the lowest at pH 2 [31].

The effect of pH on the heat-induced gelation properties of myofibrillar protein from porcine sources was observed at pH 5.6, 6.0, 6.5, and 7.0. The solutions were heated to 85 °C for 35 min and then cooled. The results showed that the decreasing pH increased the G′, with a maximum value of 1860 Pa observed at pH 5.6 [42]. The researchers compared the gelation properties of 10% (*w*/*v*) bovine gelatin with that of fish gelatin and measured gel rheology at pH values ranging from 2 to 12. It was concluded that the gel strength of both bovine and fish gelatins was dependent on the pH within the range of pH 4–10. G′ initially increased until pH 5, at which it remained stable until pH 7. It then increased again until pH 10, at which it began to decrease [26].

### 2.2. Plant Proteins

The heat-induced gelation of soy protein globulins was investigated over pH values ranging from 5.8 to 6.8 and temperatures from 65 to 95 °C. The stiffness of soy protein isolate (SPI) gels was found to be largely independent of the pH. However, the protein aggregation and gelation rate increased as the pH decreased [32]. In another study, the independence of the gelation properties of soy proteins was confirmed by varying the pH of SPI solutions between 5.8 and 7.0 [4]. After gel formation by heating at 80 °C with a concentration of 95 g/L, it was determined that decreasing pH resulted in a decrease in the critical gel concentration and gelation rate. The gel strength was not affected by changes in the pH. These results contrast with older studies referenced by the researchers, which found that the gel strength of a 12% (*w*/*v*) SPI solution was indeed dependent on the pH, with less-firm gels observed at pH levels below 6.0 [43].

The thermal gelation of pea proteins (12% *w*/*v*) was investigated at pH values of 2, 6.5, and 8. The pea protein concentrate exhibited poor gelling properties at pH 2.0, with G′ and loss modulus (G″) values below 5 Pa, while stronger gels were formed at the higher pH values [33]. Another study in a different pH range showed that, at pH 4.5, pea protein formed the stiffest gels, demonstrating a high G′ and a low tan δ, indicating strong protein interactions. The solubility of pea protein was lowest at this pH, with a solubility of 50% at 0.6 M NaCl, which contributed to the formation of firm gels. At pH 9, the formation of a continuous gel network was hindered, resulting in an entangled solution instead of a well-defined gel network [34]. It is clear that the pH is an important factor in pea protein gelation. For example, pea protein isolate gels formed at acidic pH levels (2.1 and 3.9) exhibited a coarse network structure with a low WHC. In contrast, at neutral to alkaline pH levels (6.3 and 8.3), the gels demonstrated a more solid-like character with a compact, homogeneous matrix, resulting in a better WHC. The type of interactions within the gel varied with the pH, with hydrogen bonds playing a dominant role at pH 6.3 and 8.3 [35]. Heat-induced gels were formed from pea protein that was salt-extracted from pea flour. The maximum stiffness was observed at pH 4.0 (with 0.3 M NaCl), while higher and lower pH values resulted in less-firm gels. The pH also affected the gelation temperature, with higher pH values being associated with higher gelling temperatures and the maximum gelling temperature observed at pH 6 (89.1 °C) [36].

The gelling properties of canola protein isolate (CPI) were observed at pH values ranging from 3.0 to 9.0 and compared to those of commercial SPI. It was found that CPI did not form gels at a low pH (3.0) but exhibited increasing gel strength with rising pH, reaching maximum strength at pH 9.0. The gelling properties of CPI were found to be comparable to those of SPI [44]. Overall, the plant-based and animal-based proteins observed had minimum solubility around their pI. When there is a net-zero charge, proteins will be attracted to each other burying their hydrophilic residues inside thereby reducing hydration in an aqueous solvent. Protein solubility increased as the pH moved further away from the pI. As the proteins gained a charge, electrostatic repulsion and hydrogen bonding with water occurred. While casein and egg proteins had the highest solubility, the high solubility of plant proteins such as pea, soy, sunflower seed, and wheat could be achieved by the alteration of the pH.

## 3. Effect of Ionic Environment on Gelation Properties

### 3.1. Animal Proteins

In NaCN samples with added NaCl, the gel formed at a lower pH compared to samples without NaCl. Additionally, G′ was lower at pH 4.6 in the presence of NaCl than in samples without NaCl [18]. The effect of added NaCl on the heat-induced gelation of WPI was tested by dispersing WPI in a solution at pH 6.7 with either 60 or 100 mM NaCl. The rheological properties were measured using a controlled stress rheometer with temperature control. The G′, G″, and phase angle (δ) were recorded during the heating. The concentration of protein required to form a gel was determined to be 1.8% for samples without NaCl and 1.1% for those with 100 mM NaCl, indicating that a higher concentration of NaCl reduces the amount of protein needed to form gels [25]. It is reported that NaCl, CaCl_2_, MnCl_2_, and ZnCl_2_ each impact the gelation of whey proteins through distinct mechanisms. CaCl_2_ improved gelation and increased viscosity in processed whey protein, whereas NaCl did not alter the viscosity. Inorganic salts like CaCl_2_ created firmer gels compared to organic salts, and higher salt concentrations (20 mM) led to reduced gel strength and WHC. Reheating the gels enhanced firmness but diminished adhesiveness and slightly affected the transparency and cohesiveness [45]. Divalent cations like CaCl_2_ (7.5 mM or higher), MnCl_2_, and ZnCl_2_ enhance whey protein gelation by increasing gel strength. CaCl_2_ forms firm, opaque gels with a low shear strain, while MnCl_2_ and ZnCl_2_ similarly improve the gel strength but with varying textural effects. Divalent cations overall produce stronger, more cohesive gels compared to monovalent cations [46].

In thermally induced egg white protein gels, high concentrations of NaCl (120 mM) resulted in maximum water release from the gel across all pH levels [29]. Egg white and whole egg treated with 6% NaCl exhibited lower hardness at all pH values, while egg yolks showed higher hardness with the addition of 6% NaCl. Also, the addition of 6% NaCl increased the gelation point for all egg proteins [31]. The addition of salt at concentrations of 3% and 6% (*w*/*w*) influenced the gel’s mechanical properties and WHC. Salt increased the firmness and elasticity but significantly reduced the WHC [30].

The effect of salt on the gelation of gelatin from porcine skin was investigated. Gels were formed by hydrating gelatin powder to a concentration of 5% (*w*/*v*) and adding varying levels of NaCl (0.1, 0.05, 0.02, and 0.01 M). The samples were held overnight at room temperature and then heated at five different temperatures: 25 °C, 20 °C, 15 °C, 10 °C, and 5 °C. It was found that the G′ decreased as the salt concentration increased, resulting in a softer gel [28].

### 3.2. Plant Proteins

The addition of NaCl to soy protein solutions in varying concentrations resulted in an increase in gelation time; however, the stiffness of the gels was not affected [32]. The effect of NaCl on the heat-induced gelation properties of pea protein (14.5% *w*/*v*) was investigated by varying salt concentrations from 0.0 to 2.0 M NaCl. No crossover of G′/G″ was observed, indicating that the gel retained its primarily solid-like characteristics even at high salt concentrations [32]. The researchers noted no effect on gel stiffness as the salt concentration increased. Interestingly, without added salt, the G′ was very low, suggesting that a gel could not be formed. Syneresis was observed at salt concentrations above 0.8 M, with the most optimal gel formation occurring at concentrations between 0.3 and 0.8 M [37]. It was found that the gelation temperature increased with rising salt concentration. Additionally, at high ionic strengths (0.9 M and 1.5 M NaCl) and pH 9, pea protein formed an entangled solution rather than a gel, as the denaturation temperature exceeded the gelation temperature [31]. However, at lower pH levels (2.1 and 3.9), increasing the ionic strength (μ = 0.54) enhanced the gelation of pea protein isolate. The gels formed under these conditions had improved texture properties and WHC. The higher ionic strength reduced electrostatic repulsion, thereby reinforcing the protein–protein interactions and contributing to a more solid-like gel network [35].

The effect of NaCl concentration on wheat bran protein (WBP) was investigated by altering the NaCl content in aqueous WBP solutions, specifically using 0.1 M NaCl. As mentioned previously, WBP was unable to form a heat-set gel in a deionized (DI) water suspension. However, the addition of NaCl improved the gelation capacity of the protein. Very strong gels were formed at a minimum protein concentration of 16% (*w*/*v*) [38].

The heat-set gelation of CPI was investigated with the addition of 0.1 and 0.5 M NaCl. The pH of the solution was adjusted to 7.0, and three different protein concentrations were tested (5.0%, 7.0%, and 9.0% *w*/*w*). Rheological measurements were performed using a temperature-controlled plate rheometer. It was found that there was no significant effect on gel formation and strength with the addition of either 0.1 or 0.5 M NaCl [40].

## 4. Effect of Temperature on Gelation Properties

### 4.1. Animal Proteins

The effects of ohmic heating on NaCN gels were studied by preparing gels through acidification using GDL with 6% protein. The samples were heated at 95 °C for 15 min using two heating methods (conventional and ohmic) and then cooled in an ice bath. The rupture stress and strain, which are indicative of gel hardness, were significantly different for the heated samples compared to those for the control. The heated gels exhibited greater syneresis than the unheated samples [20]. The higher syneresis observed in heated gels was primarily attributed to the structural changes in proteins induced by heating, which adversely affected the gel’s network structure and its ability to retain water. Furthermore, the application of an electric field during the ohmic treatment further modified the protein solubility and the formation of the gel network, leading to an increased water loss observed as syneresis [20].

WPI gels were formed by initially heating to 50 °C for 15 min, followed by further heating to 60, 65, 70, 75, 80, 85, and 90 °C for 15 min at each temperature. The gels were then cooled to 4 °C for 15 h and brought to room temperature before testing. It was determined that, as the temperature increased, irreversible denaturation occurred, resulting in increased polymerization of the whey proteins [23]. Hence, heating enhanced the polymerization of whey protein gels, resulting in gels with proteins that have a greater molecular weight. The degree of polymerization and gel formation was studied, with more significant effects observed at higher pH levels (pH 9 and 11) and lower temperatures [23]. Similarly, heating milk protein concentrates at 85 °C for 5 min resulted in more whey protein denaturation and increased gel firmness compared to heating at 125 °C for 15 s. The gels formed at 85 °C demonstrated higher viscosity and firmness, indicating that lower temperatures for longer durations favor the formation of stronger gels [24]. In both studies, higher temperatures led to increased protein denaturation and polymerization, which impacted the gel structure.

The gelation properties of white and red chicken myofibrillar proteins (CMPs) were observed after 60 min of heating at temperatures ranging from 20 °C to 60 °C. The G′ was consistently greater than the G″, indicating a predominantly elastic gel. As the temperature increased, the G′ gradually increased as well [27].

### 4.2. Plant Proteins

The effect of thermal treatment on the gelation of SPI was investigated by varying the gelation temperature between 65 °C and 85 °C. The rheological characteristics of the gel (at a concentration of 95 g/L and pH 6.0) were measured using a rheometer. It was found that the gelation time decreased with increasing temperature, showing an approximately linear relationship. After normalizing the data to account for heating time and gel time, there was no significant difference in the G′ as a function of temperature [4].

The heat treatment of pea proteins resulted in poorer gelling properties. Samples of pea protein isolate (PPI) were heated from 25 °C to 95 °C and then cooled before testing. The heat-treated proteins formed gels more slowly, requiring higher temperatures for gelation to occur. The strength of the resultant gels was reduced, as indicated by lower G′ values [37]. Also, pea protein did not form significant gels at temperatures above 95 °C. The higher temperatures led to increased denaturation temperatures and incomplete protein interactions, resulting in an entangled solution rather than a solid gel network [34].

Rapeseed protein isolate gels were subjected to heat and pressure treatment at temperatures of 60 °C, 80 °C, and 100 °C. Texture analysis revealed that heating gels from 60 °C to 80 °C resulted in increased hardness, adhesiveness, springiness, and cohesiveness. However, these properties were reduced when the gel was heated to 100 °C [41].

The heat-induced gelation of gluten protein was affected by the treatment temperature. Dispersions of gluten powder at pH 7.0 were incubated at various temperatures between 25 °C and 90 °C for 30 min before observing their rheological behavior. A reduction in G′ was observed with increasing temperature from 25 °C to 60 °C, after which G′ began to increase from 60 °C to 90 °C. These measurements indicate that weaker gels are formed during the initial stages of denaturation, while stronger gels result as the proteins become more denatured. However, the gels formed at higher temperatures were still weaker than those formed at the initial gelation temperature of 25 °C [39].

## 5. Effect of Anti-Nutritional Factors on Gelation Properties

Several compounds have been identified in plant protein sources that affect protein digestibility, known as anti-nutritional factors (ANFs). These include compounds such as phytic acid, tannins, saponins, lectins, and enzyme inhibitors. While these ANFs influence nutritional value, they also affect the functionality of plant proteins in food systems [47]. Also, these ANFs reduce amino acid availability, alter protein structures, and block essential functional groups, leading to weaker and less-stable gels [48,49]. Hence, lectins, saponins, and tannins have minimal or no direct impact on the gelation properties of animal proteins. Consequently, animal-based proteins generally exhibit more robust gelation properties and are less affected by ANFs.

### Plant Proteins

The heat-set gelation of soy proteins was affected by the presence of ANFs. Rheological measurements of SPI gels in the presence of phytic acid, tannic acid, and Quillaja bark saponin (QBS) were conducted. At pH 3.0, the presence of phytic acid and QBS resulted in increased gel strength compared to the control, while tannic acid dramatically reduced the gel strength during initial heating. A drop in the G′ was observed for SPI gels with phytic acid and tannic acid after cooling to 20 °C [47].

The presence of ANFs such as phytic acid, tannic acid, and QBS significantly affected the heat-set gelation of PPI. At pH 3.0, all ANFs resulted in a reduction in the G′ at all stages of gelation. However, at pH 7.0, tannic acid and QBS increased the G′ during the initial heating stage. After cooling, the G′ was reduced for samples containing phytic acid and tannic acid, while it increased in the presence of QBS [47].

Rice protein isolate gelation was influenced by the presence of ANFs. In gels adjusted to pH 3.0, the addition of phytic acid, tannic acid, or QBS led to a reduction in the G′ during both the initial heating process and after complete cooling. However, samples containing QBS exhibited an increase in the G′ in the middle of heating at 90 °C. At pH 7.0, gels with QBS and phytic acid showed increased G′ at the outset of heating. During the middle of the heating process, gels with QBS continued to display increased G′, while at the end of heating, samples with phytic acid experienced an increase in the G′ compared to those without ANFs [47].

The interaction of several phenolic compounds with canola protein in heat-set gels was investigated. It was determined that both compounds tested, sinapic acid and thomasidioic acid, negatively affected gelation, as measured by dynamic rheology, at all concentrations and pH conditions tested [50].

Overall, animal-based proteins contain a significantly lower amount of ANFs than plant-based proteins and require less processing to improve their protein quality. Common methods of removal include heat treatments, fermentation, and physical processes. Soaking is effective in removing water-soluble ANFs such as phytic acid, tannins, and saponins; however, methods other than soaking should be considered when removing ANFs as soaking can also reduce the protein content of foods due to the fact that proteins are water-soluble molecules. Choosing cultivars with lower ANFs is also beneficial when considering protein quality. Examining and adapting methods for removing or reducing ANFs are essential as plant products with limited protein digestibility such as legumes, cereals, and grains are the primary protein sources in developing countries [51].

## 6. Effect of Blends on Gelation Properties

Mixing animal- and plant-based proteins is a rapidly emerging area of food science, driven by the need for sustainable alternatives to traditional animal proteins and the growing consumer demand for plant-based options [52]. A recent study explored the gelation properties of SPI and WPI. The researchers discovered that when SPI and WPI were mixed and heated, they formed gels with unique network structures that varied with their concentrations. At higher SPI concentrations, the soy proteins formed a continuous gel network, while at higher WPI concentrations, the whey proteins took over the gel formation. That study identified an optimal SPI to WPI ratio of 85:15, beyond which the gels became unstable and susceptible to syneresis [53]. In another study, the gelation behavior of mixtures of soy protein concentrate (SPC) and whey protein was examined. The research demonstrated that, at a total protein content of 6%, whey proteins primarily formed the gel network. However, as the proportion of SPC increased, the gel network became more complex, with soy proteins aggregating during the cooling process, which impacted the overall gel strength and stability. Both of these studies highlighted that the concentration and ratio of soy and whey proteins significantly influenced the gelation properties [54]. In contrast, heat-induced gel studies revealed that equal parts milk and pea protein formed firmer gels than milk alone, with a similar firmness to pea protein gels. However, enzyme-mediated gelation with chymosin showed reduced gel strength due to pea protein’s interference with casein gelation. During acidification, pea and milk protein mixtures gelled more rapidly than milk alone [55]. Protein mixtures with micellar caseins and plant proteins like pea or soy did not co-aggregate but formed separate networks, with the gel strength largely dependent on the protein concentration and not on the pH. For instance, the strength decreased at higher plant protein concentrations. The critical temperature for gelation was reduced as the pH decreased. The gel strength was dependent on the plant protein concentration, with minimum strength occurring at 40% soy protein and 70% pea protein [56]. Furthermore, blending SPI with surimi significantly impacted the gelation properties. An 80:20 SPI-to-surimi ratio showed the best results in terms of chewiness, hardness, and fibrous texture. In contrast, a 60:40 ratio weakened the gel strength and extended the gelation time. Excess surimi (more than 40%) interfered with the formation of a fibrous structure. SPI acted as the continuous phase with surimi dispersed throughout, and the gel strength exhibited a nonlinear relationship [57].

## 7. Conclusions

The comparative analysis of gelation abilities in plant-based and animal-based proteins highlighted significant differences driven by factors such as the pH, ionic environment, and temperature. Whey and casein proteins formed clearer and stronger gels, which are highly desirable in many food formulations. In contrast, SPI and rice protein isolate showed greater variability in gelation abilities due to their structural complexity and the presence of ANFs. Furthermore, the ionic environment and temperature played key roles in the gelation of both plant-based and animal-based proteins, affecting the strength and texture of the final product. This review highlights the key factors involved in protein selection and processing conditions to achieve desired gel characteristics, paving the way for the optimization of food formulations.

## Figures and Tables

**Table 1:** Summary of results on the effect of pH, temperature, and ionic environment on gelation properties of animal and plant proteins.

Protein	Parameter	Effect	Reference
Casein 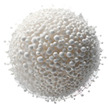	pH	No effect	[19]
	Temperature	Heating → greater syneresis	[20]
	Ionic env. *	Gel formation at lower pH with NaCl added than without	[18]
Whey 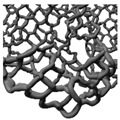	pH	Lowest at pI, optimum pH variable	[21,22]
	Temperature	Heating → increased polymerization 85 °C for 5 min: ↑ whey protein denaturation, ↑ viscosity, ↑ gel firmness 125 °C for 15 s: ↓ whey protein denaturation, ↓ viscosity, ↓ gel firmness	[23] [24]
	Ionic env.	Less protein required to form a gel with 100 mM NaCl added	[25]
Meat 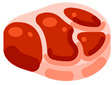	pH	↑ pH ↓ G′	[26]
	Temperature	↑ Temp ↑ G′	[27]
	Ionic env.	↑ NaCl ↓ G′	[28]
Egg 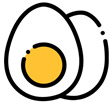	pH	Coarse gel at pI, optimum at pH 7 and 9 ↓ pH near 4.5 (isoelectric point of ovalbumin): ↓ gel firmness, ↓ WHC **, brittle gel formation pH 6.5–8.0: better gel structure	[29] [30]
	Temperature	↑ pH ↑ gelation temp	[31]
	Ionic env.	↑ NaCl ↓ hardness (EW, WE), ↑ NaCl ↑ hardness (EY), ↑ NaCl ↑ gelation time ↑ Firmness, ↑ elasticity, ↓ WHC	[31] [30]
Soy 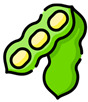	pH	No effect on G′, ↑ gel rate ↓ pH	[32]
	Temperature	No significant difference	[4]
	Ionic env.	No effect on stiffness, ↑ NaCl ↑ gelation time	[32]
Pea 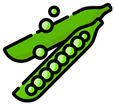	pH	↑ pH ↑ G′ Stiffest gels at pH 4.5 with 0.6 M NaCl Gel stiffness ↓ at higher pH ↓ pH 2.1, 3.9: ↓ WHC, ↓ gelation ↑ pH 6.3, 8.3: ↑ WHC, ↑ firmness	[33] [34] [35]
	Temperature	↑ pH ↑ gelation temp, ↑ temp ↓ G′ No significant gelation beyond 95 °C	[36,37] [34]
	Ionic env.	No effect on stiffness, ↑ NaCl ↑ gelation time ↑ Ionic strength →↑ denaturation temp ↑ Ionic strength →↑ gel formation	[36] [34] [35]
Wheat 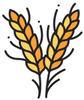	pH	No gel formation without NaCl	[38]
	Temperature	↑ Temp ↓ G′ (to 60 °C), ↑ Temp ↑ G′ (60–90 °C)	[39]
	Ionic env.	↑ NaCl ↑ gelation capacity	[38]
Oilseed 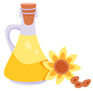	pH	No gel at pH 3, ↑ pH ↑ G′ (max at pH 9)	[40]
	Temperature	↑ Temp ↑ hardness, springiness, etc.	[41]
	Ionic env.	No effect	[40]

* Ionic environment. ** Water-holding capacity (WHC).

## Data Availability

No new data were created or analyzed in this study. Data sharing is not applicable to this article.
